# Cigarette smoking and breast cancer risk in relation to joint estrogen and progesterone receptor status: a case-control study in Japan

**DOI:** 10.1186/2193-1801-3-65

**Published:** 2014-02-03

**Authors:** Yoshikazu Nishino, Yuko Minami, Masaaki Kawai, Kayoko Fukamachi, Ikuro Sato, Noriaki Ohuchi, Yoichiro Kakugawa

**Affiliations:** Division of Cancer Epidemiology and Prevention, Miyagi Cancer Center Research Institute, 47-1 Nodayama, Medeshima-Shiode, Natori, Miyagi, 981-1293 Japan; Division of Community Health, Tohoku University Graduate School of Medicine, 2-1 Seiryo-machi, Aoba-ku, Sendai, Miyagi, 980-8575 Japan; Department of Surgical Oncology, Tohoku University Graduate School of Medicine, 1-1 Seiryo-machi, Aoba-ku, Sendai, Miyagi, 980-8574 Japan; Department of Breast Oncology, Miyagi Cancer Center Hospital, 47-1 Nodayama, Medeshima-Shiode, Natori, Miyagi, 981-1293 Japan; Department of Pathology, Miyagi Cancer Center Hospital, 47-1 Nodayama, Medeshima-Shiode, Natori, Miyagi, 981-1293 Japan

**Keywords:** Breast cancer, Smoking, Passive smoking, Hormone receptor, Menopausal status, Case-control study

## Abstract

An association of cigarette smoking with breast cancer risk has been hypothesized. However, results from previous studies have been inconsistent. This case-control study investigated the association of cigarette smoking with breast cancer risk in terms of estrogen-receptor/progesterone-receptor (ER/PgR) status. From among female patients aged 30 years and over admitted to a single hospital in Japan between 1997 and 2011, 1,263 breast cancer cases (672 ER+/PgR+, 158 ER+/PgR-, 22 ER-/PgR+, 308 ER-/PgR- and 103 missing) and 3,160 controls were selected. History of smoking (ever, never), some smoking-related measures, and passive smoking from husbands (ever, never) were assessed using a self-administered questionnaire. Polytomous logistic regression and tests for heterogeneity across ER+/PgR + and ER-/PgR- were conducted. For any hormone receptor subtype, no significant association was observed between history of smoking (ever, never) and breast cancer risk. Analysis of smoking-related measures revealed that starting to smoke at an early age of ≤19 years was significantly associated with an increased risk of postmenopausal ER-/PgR- cancer (odds ratio = 7.01, 95% confidence interval: 2.07-23.73). Other measures of smoking such as the number of cigarettes per day, the duration of smoking, and start of smoking before the first birth were not associated with breast cancer risk for any receptor subtype. There was no association between passive smoking (ever, never) and breast cancer risk for any of the four subtypes. These results indicate that history of smoking and passive smoking from husbands may have no overall effect on breast cancer risk for any hormone receptor subtype. However, it is possible that women who start to smoke as teenagers may have a higher risk of developing postmenopausal ER-/PgR- cancer. Further studies are needed to clarify the association of smoking with breast cancer risk, especially the role of starting to smoke at an early age.

## Introduction

Breast cancer is one of the most common cancers worldwide (Curado et al. 2007). Although Japan has a lower risk of breast cancer in comparison with Western countries, its incidence is first in terms of age-standardized rates among all female cancers, and it is increasing continuously (Matsuda et al. 2012; Minami et al. 2004). The established risk factors for breast cancer include menstrual and reproductive history, family history of breast cancer, some anthropometric measures such as tallness and postmenopausal obesity, and alcohol consumption (Kelsey et al. 1993; Kawai et al. 2010; Pharoah et al. 1997; Renehan et al. 2008; van den Brandt et al. 2000; Key et al. 2006). Most of these risk factors are not preventable.

Cigarette smoking is known to be a preventable risk factor for cancer including several major sites such as the lung and stomach (Minami and Tateno 2003; Katanoda et al. 2008). An association of cigarette smoking with breast cancer risk has also been hypothesized (Palmer and Rosenberg 1993), and numerous epidemiologic studies have investigated this issue. However, the results have been less consistent compared with those for other smoking-related cancers. In Western countries, a pooled analysis of 53 epidemiologic studies (Hamajima et al. 2002) has showed that smoking was not associated with breast cancer risk, whereas a review by Terry and Rohan suggested an increased risk of breast cancer among women with specific characteristics such as smoking of long duration (Terry and Rohan 2002). A recent report from Canada indicated a positive association between active smoking and breast cancer risk (Johnson et al. 2011). The IARC Monograph in 2012 suggests the positive association, although evidence is limited (International Agency for Research on Cancer 2012; Secretan et al. 2009). In Japan, a systematic review including three cohort studies and eight case-control studies concluded that smoking possibly increases breast cancer risk (Nagata et al. 2006); however, only a few studies have demonstrated a significant association between smoking and an increased risk of breast cancer (Hanaoka et al. 2005).

From the viewpoint of biological mechanisms, the relationship between cigarette smoking and the development of breast cancer is likely a complex one. Tobacco smoke contains potential human breast carcinogens such as polycyclic aromatic hydrocarbons, aromatic amines, and N-nitrosamine (Zaridze and Peto 1986). These carcinogens may induce mammary tumors (Hecht 2002). Conversely, smoking has been postulated to have an antiestrogenic effect, which may also affect the development of breast cancer (Baron 1984; Tanko and Christiansen 2004). Taking into account these biological characteristics of cigarette smoke, its association with breast cancer risk may differ according to menopausal status and hormone receptor status. However, most studies have evaluated the overall association between cigarette smoking and the risk of breast cancer, which may account for the inconsistency in the results mentioned above. Few studies have focused on breast cancer risk with reference to hormone receptor status (Yoo et al. 1997; Manjer et al. 2001; Gammon et al. 2004; Luo et al. 2011).

To clarify the association of cigarette smoking with breast cancer risk according to menopausal status and hormone receptor status, we conducted a hospital-based case-control study. Data were obtained from women aged 30 years and over who were admitted to a single hospital in Miyagi Prefecture, Japan, between 1997 and 2011. Analyses were performed with reference to joint estrogen-receptor/progesterone-receptor (ER/PgR) status, i.e., ER+/PgR+, ER+/PgR-, ER-/PgR+, and ER-/PgR-.

## Methods

### Data collection

In January 1997, we began a questionnaire survey in connection with the present study. Information on lifestyle and personal history was collected from all patients at their first admission to the Miyagi Cancer Center Hospital (MCCH), using a self-administered questionnaire. The questionnaire was distributed to patients on the day of their reservation for initial admission, i.e., 10-15 days before admission, and collected by nurses on the day of actual admission. The purpose of the survey was stated on the cover page of the questionnaire. We considered the return of self-administered questionnaires signed by the patients to imply their consent to participate in the study.

The MCCH is located in the southern part of Miyagi Prefecture, and functions as a hospital for both cancer and benign diseases. Details of the questionnaire survey have already been described elsewhere (Minami and Tateno 2003; Minami et al. 2012; Kawai et al. 2012; Seki et al. 2013).

The questionnaire covered demographic characteristics, personal and family histories of cancer and other diseases including a family history of breast cancer in mother or sisters, general lifestyle factors before the development of current symptoms, including cigarette smoking, alcohol drinking, exercise, occupation, menstrual and reproductive histories, and histories of oral contraceptive (OC) use and exogenous female hormone use. Items related to the referral status and area of residence were also included. Between January 1997 and December 2011, the questionnaire was distributed to 26,984 first-admitted patients, of whom 24,062 responded.

### Study subjects

Cases and controls were selected from among 24,062 patients who responded to the questionnaire survey. To identify incident cases of female breast cancer, a list of the patients was linked with both the hospital-based cancer registry file and the disease registration database at MCCH. The cancer registry records all cancer cases confirmed by clinical, cytological and/or histopathological examination at the MCCH. Through the linkage, 24,062 patients were classified into 2,219 with a past history of cancer, 7,707 males with cancer, 1,309 females with breast cancer, 4,779 females with other cancers, and 8,048 non-cancer patients (4,170 males and 3,878 females). Among the 1,309 females with breast cancer, 1,302 aged 30 years and over were included as the study cases.

Controls were selected from among female non-cancer patients without history of cancer (n = 3,878). Patients with benign tumors have been classified as non-cancer patients for the present study. After excluding patients under 30 years, 3,587 female non-cancer patients aged 30 years and over were selected as possible controls, from whom 295 subjects with smoking-related disease were excluded. Finally, a total of 3,292 female non-cancer patients were included as controls. Smoking-related diseases were defined based on the procedure used in our previous studies (Minami and Tateno 2003; Seki et al. 2013). The diagnoses among the 295 excluded subjects were heart disease in 118, respiratory disease in 130, and benign tumors including the upper respiratory tract in 18, esophagus in 10 and urinary tract in 19. The diagnoses among the 3,292 controls were as follows: benign tumor 2,083 (63.3%), digestive tract disease 403 (12.2%), urologic-gynecologic disease 176 (5.4%), endocrine or metabolic disease 87 (2.6%), orthopedic disorder 54 (1.6%), other benign disease 207 (6.3%), and no abnormal findings 282 (8.6%). The sites of benign tumors were stomach in 156 subjects, colorectum in 532, lung in 21, breast in 44, gynecologic organs in 381, bone or connective tissue in 636, and other in 313.

This study was approved by the ethical review board of the Miyagi Cancer Center.

The final response rate for the questionnaire survey was 94.4% for the cases and 89.6% for the possible controls.

### Assessment of cigarette smoking

Information on exposure, i.e., cigarette smoking, was collected from the above questionnaire survey. Exposure variables related to active smoking included history of smoking (never-, past-, current-smoker), age at the start of smoking (never, ≤19, ≥20- ≤ 25, ≥26 yr), whether smoking had started before the first birth (never, no, yes, uncertain), duration of smoking (never, >0- ≤ 21, >21), the mean number of cigarettes smoked per day during the smoking period (never, >0- ≤ 10, ≥11 yr), and the number of pack-years of smoking (never, >0- ≤ 13, >13). Subjects who quit smoking within one year before the present admission were regarded as current smokers. Pack-years of smoking were calculated by multiplying the duration of smoking by the mean number of cigarettes smoked per day divided by 20. Whether smoking had started before the first birth was defined by comparing the age at the start of smoking and the age at first birth. When age at the start of smoking was equal to the age at first birth, the case was assigned to the “uncertain” category. Cut-off points for age at the start of smoking were determined arbitrarily, considering the importance of the category for subjects who started smoking as teenagers. For the other three exposure variables, the mid-point among the controls was used as a cut-off point. Subjects for whom data on smoking history were missing [n = 39 (3.0%) for cases and n = 132 (4.0%) for controls] were excluded from the subsequent analysis, leaving 1,263 cases and 3,160 controls.

The only exposure variable related to passive smoking was the husband’s smoking status (never-, past-, current-smoker). This information was obtained from married subjects. The risk for passive smoking was investigated only among never-smokers.

### Hormone receptor status

Information on the expression of ER and PgR in breast cancers was extracted from medical records. To measure ER/PgR status, enzyme immunoassay (EIA) had been used in the early period of the study to determine hormone receptor status. After mid-2003, immunohistochemistry (IHC) assay was routinely conducted. The concordance between the two assays was 94.3% for ER and 100% for PgR in the laboratory of the MCCH (Kakugawa et al. 2007).

### Statistical analysis

We used multiple polytomous unconditional logistic regression analysis to estimate hormone receptor-defined breast cancer risk. In the analysis, study subjects were categorized using the cut-off points for each exposure and odds ratios (ORs) and 95% confidence intervals (CIs) were calculated for each category.

We considered the following variables to be potential confounders: age, year of recruitment, referral status (from screening, other), area of residence (Southern Miyagi prefecture, others), alcohol drinking (ever, never), occupation (professional or clerical work, industrial work or fishery, agriculture or forestry, other), age at menarche, age at menopause, menopausal status (premenopausal, postmenopausal), reason for menopause (natural menopause, menopause for other reasons), parity number, age at first birth, family history of breast cancer in mother or sisters (yes, no), physical activity (almost no, more than one hour per week), body mass index (BMI), and history of use of exogenous female hormones or OCs (ever, never). Occupation classification was based on the categorization used in our previous study (Seki et al. 2013). Missing values were treated as an additional variable category.

We stratified case subjects according to joint hormone receptor status: ER+/PgR+, ER+/PgR-, ER-/PgR+, and ER-/PgR-. Stratification by menopausal status was also performed. Menopause was defined as the cessation of menstrual periods due to natural or other reasons, including surgery (Kawai et al. 2012). In the analysis stratified by menopausal status, we excluded cases with ER+/PgR- or ER-/PgR + tumors because these were too few to allow precise estimation of ORs in comparison with subjects who had ER+/PgR + or ER-/PgR- tumors.

Dose-response relationships were tested by treating each exposure category as a continuous variable. We conducted Wald tests for heterogeneity of breast cancer risk across ER+/PgR + and ER-/PgR-. Values were regarded as significant if the two-sided *P* values were <0.05. All analyses were performed using SAS version 9.3 (SAS Institute, Cary, NC).

## Results

The baseline characteristics of the study subjects are presented in Table 1. Joint ER/PgR status was available for 1,160 cases (91.8%): 672 were ER+/PgR+, 158 were ER+/PgR-, 22 were ER-/PgR+, and 308 were ER-/PgR-. Cases with ER-/PgR- tumors were less likely to have been referred from screening. Those with ER+/PgR + tumors were more likely to be heavier, and to be nulliparous.

**Table 1:** **Characteristics of cases and controls by hormone receptor status**

	Cases					Controls
	Hormone receptor					
	ER+/PgR+	ER+/PgR-	ER-/PgR+	ER-/PgR-	Missing	
Number of subjects	672	158	22	308	103	3160
Age group (years old) (%)						
30-39	6.4	3.2	9.1	4.9	9.7	9.7
40-49	26.5	17.1	45.5	22.4	12.6	17.8
50-59	27.8	28.5	22.7	30.8	35.0	22.1
60-69	22.9	32.9	13.6	23.1	18.5	25.2
70 ≤	16.4	18.4	9.1	18.8	24.3	25.3
Mean (years old)	56.7	59.5	52.3	57.8	58.7	59.0
SD	12.4	11.1	11.7	12.1	13.2	13.9
Menopausal status (%)^a^						
Premenopausal	41.1	22.2	45.5	29.9	23.3	29.6
Postmenopausal	54.4	74.0	40.9	63.3	46.6	65.1
Unknown menopausal status	4.5	3.8	13.6	6.8	30.1	5.3
Year of recruitment (%)						
1997-2003	30.1	38.0	54.6	42.9	52.4	52.7
2004-2011	69.9	62.0	45.5	57.1	47.6	47.3
Referrel status (%)						
From screening	21.7	20.3	18.2	15.3	7.8	18.2
Other	78.3	79.8	81.8	84.7	92.2	81.8
Area of residence (%)						
Southern Miyagi prefecture	83.5	85.4	81.8	82.8	79.6	88.3
Other area	16.5	14.6	18.2	17.2	20.4	11.7
Occupation (%)						
Agriculture or forestry	5.1	3.8	9.1	6.2	6.8	9.1
Industrial work or fishery	16.5	12.7	9.1	19.8	10.7	14.3
Professional or clerical work	45.2	46.8	40.9	37.7	33.0	36.0
Other^b^	22.2	26.6	27.3	23.7	30.1	24.5
Missing	11.0	10.1	13.6	12.7	19.4	16.0
BMI (kg/m2, %)						
< 18.5	5.1	6.3	-	5.2	9.7	5.9
18.5 ≤ < 25.0	59.4	63.9	59.1	66.6	62.1	63.9
25.0 ≤ < 30.0	26.8	24.7	40.9	22.4	21.4	25.3
30.0 ≤	8.3	5.1	-	5.5	3.9	4.0
Missing	0.5	-	-	0.3	2.9	1.0
Mean	24.0	23.5	24.2	23.3	23.0	23.4
SD	3.8	3.7	3.2	3.9	3.5	3.5
Time spent exercising (%)						
Almost no	50.5	49.4	45.5	51.3	50.5	48.6
1≤ hr per week	44.1	43.7	50.0	41.6	38.8	45.2
Missing	5.5	7.0	4.6	7.1	10.7	6.2
Age at menarche (%)						
≤ 12	33.8	20.9	36.4	30.2	28.2	23.3
13	22.8	21.5	22.7	22.1	20.4	19.9
14	19.1	27.9	18.2	20.8	16.5	18.9
15 ≤	20.8	25.3	18.2	22.7	26.2	30.8
Missing	3.6	4.4	4.6	4.2	8.7	7.1
Age at menopause (%)						
< 48	12.3	6.0	22.2	11.8	18.8	12.5
48 ≤ < 51	30.6	29.9	22.2	24.1	41.7	26.9
51 ≤ < 54	18.6	27.4	33.3	25.1	27.1	19.7
54 ≤	13.9	15.4	11.1	12.3	12.5	8.7
Missing	24.6	21.4	11.1	26.7	0.0	32.2
Parity number (%)						
0	12.1	7.6	4.6	6.2	11.7	7.9
1	10.4	9.5	18.2	13.3	8.7	9.1
2	45.5	43.7	59.1	44.8	42.7	40.8
3 ≤	24.4	29.1	9.1	28.6	27.2	33.3
Missing	7.6	10.1	9.1	7.1	9.7	9.1
Age at first birth (%)						
≤ 24	31.9	38.6	9.1	37.7	36.9	41.1
25 ≤ ≤ 29	39.1	36.7	59.1	39.0	35.9	35.8
30 ≤ ≤ 50	11.2	11.4	18.2	11.0	6.8	7.3
Missing	17.9	13.3	13.6	12.3	20.4	15.9
Use of exogenous female hormone or oral contraceptives (%)						
Never	81.1	79.8	90.9	83.1	76.7	78.7
Ever or current	9.8	12.0	90.9	9.7	11.7	9.1
Missing	9.1	8.2	90.9	7.1	11.7	12.3
Family history of breast cancer in mother or sister (%)						
No	90.8	87.3	81.8	88.6	94.2	96.0
Yes	9.2	12.7	18.2	11.4	5.8	4.0
Alcohol drinking (%)						
Never	67.6	79.8	77.3	70.5	75.7	72.6
Current or past	30.1	20.3	18.2	27.3	18.5	24.4
Missing	2.4	-	4.6	2.3	5.8	3.0
Smoking status (%)						
Never	82.6	81.7	77.3	83.1	82.5	83.2
Current	12.5	11.4	13.6	12.3	12.6	12.9
Past	4.9	7.0	9.1	4.6	4.9	3.9
Passive smoking (%)						
Number of subjects^c^	499	116	17	241	77	2405
Never	30.1	31.0	41.2	30.3	23.4	30.9
Past	23.9	21.6	-	19.9	14.3	20.3
Current	35.3	31.0	52.9	39.0	48.1	34.4
Missing	10.8	16a.4	5.9	10.8	14.3	14.5

^a^Menopause was defined as the cessation of menstrual periods due to natural or other reasons including surgery.

^b^Household wife/Domestic help/Student/Other.

^c^Husband’s smoking among never-smoking married women.

Table 2 shows ORs and 95% CIs for exposure variables related to active smoking according to the four hormone receptor subtypes. No association between history of smoking (ever, never) and breast cancer risk was observed for either ER+/PgR + or ER-/PgR- type. For both ER+/PgR- and ER-/PgR + types, the OR for past smoking exceeded one; however, statistical test showed that this was not significant. Age at the start of smoking was not significantly associated with breast cancer risk for any of the subtypes, although the risk for the ER-/PgR + type was not fully evaluated due to the small number of cases. An increased risk for starting to smoke at an earlier age (OR = 1.55, 95%CI: 0.76-3.20 for ≤19 years) was observed for the ER-/PgR- type, but this was not statistically significant. There was no association between having started smoking before the first birth and breast cancer risk for any of the subtypes. Analyses of the number of cigarettes smoked per day, the duration of smoking, and the number of pack-years demonstrated no significant association between these exposure variables and the risk for any subtype of breast cancer.

**Table 2:** **Odds ratio (OR) according to smoking status by hormone receptor status**

	ER+/PgR+ (n = 672)							ER+/PgR- (n = 158)						ER-/PgR+ (n = 22)						ER-/PgR- (n = 308)						***P***_***heterogeneity***_
	Controls	Cases	OR	95% CI			p	Cases	OR	95% CI			p	Cases	OR	95% CI			p	Cases	OR	95% CI			p	ER+/PgR+ vs ER-/PgR-
History of smoking																										
Never smoker	2629	555	1 (Reference)					129	1 (Reference)					17	1 (Reference)					256	1 (Reference)					
Current	408	84	0.86	0.65	-	1.14		18	0.99	0.58	-	1.69		3	1.06	0.28	-	4.12		38	0.84	0.57	-	1.23		
Past	123	33	0.92	0.60	-	1.41		11	1.80	0.92	-	3.53		2	2.53	0.49	-	12.96		14	1.02	0.57	-	1.83		
Ever	531	117	0.88	0.68	-	1.12		29	1.20	0.76	-	1.88		5	1.40	0.45	-	4.32		52	0.88	0.63	-	1.24		0.96
Age at the start of smoking (years)																										
Never smoker	2629	555	1 (Reference)					129	1 (Reference)					17	1 (Reference)					256	1 (Reference)					
26 ≤	166	28	0.85	0.55	-	1.31		10	1.22	0.61	-	2.43		0	-	-	-	-		13	0.73	0.40	-	1.33		
20 ≤ ≤ 25	253	63	0.90	0.65	-	1.24		12	1.01	0.52	-	1.94		4	2.12	0.60	-	7.51		25	0.89	0.56	-	1.41		
≤ 19	60	16	0.91	0.49	-	1.67		2	0.83	0.19	-	3.59		0	-	-	-	-		10	1.55	0.76	-	3.20		
*p for trend*							0.42						0.94						0.58						0.95	0.57
Smoking started before first birth																										
Never smoker	2629	555	1 (Reference)					129	1 (Reference)					17	1 (Reference)					256	1 (Reference)					
No	179	30	0.86	0.56	-	1.31		9	0.99	0.48	-	2.03		-	-	-	-	-		14	0.70	0.40	-	1.26		
Yes	193	53	0.95	0.67	-	1.35		12	1.36	0.70	-	2.65		4	2.30	0.63	-	8.38		23	0.97	0.60	-	1.59		
Uncertain	27	6	1.02	0.40	-	2.62		1	0.96	0.13	-	7.39		-	-	-	-	-		3	0.97	0.28	-	3.31		
Number of cigarettes per day																										
Never smoker	2629	555	1 (Reference)					129	1 (Reference)					17	1 (Reference)					256	1 (Reference)					
0 < ≤ 10	255	58	0.87	0.62	-	1.20		13	1.12	0.60	-	2.09		3	1.90	0.47	-	7.71		22	0.77	0.48	-	1.25		
11 ≤	229	51	0.92	0.65	-	1.30		11	1.01	0.52	-	1.97		1	0.61	0.07	-	5.15		25	0.99	0.63	-	1.56		
*p for trend*							0.47						0.87						0.94						0.68	0.92
Duration of smoking (years)																										
Never smoker	2629	555	1 (Reference)					129	1 (Reference)					17	1 (Reference)					256	1 (Reference)					
0 < ≤ 21	242	48	0.70	0.49	-	1.00		11	1.02	0.52	-	2.02		2	1.07	0.21	-	5.50		21	0.78	0.47	-	1.29		
21 <	226	52	1.00	0.71	-	1.40		12	1.09	0.58	-	2.06		2	1.52	0.31	-	7.41		25	1.01	0.64	-	1.59		
*p for trend*							0.49						0.80						0.63						0.77	0.85
Pack-years																										
Never smoker	2629	555	1 (Reference)					129	1 (Reference)					17	1 (Reference)					256	1 (Reference)					
0 < ≤ 13	238	48	0.71	0.50	-	1.02		12	1.18	0.61	-	2.27		3	1.67	0.41	-	6.86		17	0.63	0.37	-	1.09		
13 <	225	52	1.01	0.72	-	1.42		10	0.89	0.45	-	1.76		1	0.75	0.09	-	6.24		28	1.13	0.73	-	1.75		
*p for trend*							0.54						0.88						0.94						0.97	0.71

All models were adjusted by age (continuous), BMI (< 18.5, 18.5 ≤ < 25.0, 25.0 ≤ < 30.0, 30.0 ≤), occupation (professional or clerical work, industrial work or fishery, agriculture or forestry, other), physical activity (almost no, more than one hour per week), menopausal status (premenopausal, postmenopausal), age at menarche (≤ 12, 13, 14, 15 ≤), age at menopause (< 48, 48 ≤ < 51, 51 ≤ < 54, 54 ≤), age at first birth (≤ 24, 25 ≤ ≤ 29, 30 ≤ ≤ 50), family history of breast cancer in mother or sister (yes, no), parity number (0, 1, 2, 3 ≤), use of exogenous female hormone or oral contraceptives (yes, no), referrel status (from screening, other), year of recruitment (continuous), area of residence (Southern Miyagi prefecture, other area) and alcohol drinking (never, current or past).

Table 3 shows the results for premenopausal women according to ER+/PgR + and ER-/PgR- status. History of smoking was not associated with breast cancer risk for any of the tumor subtypes. No association with breast cancer risk was also observed for age at the start of smoking, start of smoking before the first birth, the number of cigarettes smoked per day, and duration of smoking.

**Table 3:** **Odds ratio (OR) among premenopausal women according to smoking status by hormone receptor status**

	Premenopausal													
	Controls	ER+/PgR+ (n = 276)						ER-/PgR- (n = 92)						***P***_***heterogeneity***_
		Cases	OR	95% CI			p	Cases	OR	95% CI			p	
History of smoking														
Never smoker	668	204	1 (Reference)					69	1 (Reference)					
Current	220	51	0.89	0.60	-	1.32		20	0.92	0.52	-	1.64		
Past	49	21	1.09	0.59	-	2.02		3	0.49	0.14	-	1.69		
Ever	269	72	0.94	0.65	-	1.34		23	0.82	0.48	-	1.42		0.67
Age at the start of smoking (years)														
Never smoker	668	204	1 (Reference)					69	1 (Reference)					
26 ≤	42	12	1.19	0.57	-	2.47		3	0.61	0.18	-	2.12		
20 ≤ ≤ 25	167	40	0.80	0.52	-	1.24		16	0.93	0.50	-	1.74		
≤ 19	47	15	1.12	0.56	-	2.26		4	0.83	0.27	-	2.54		
*p for trend*							0.65						0.68	0.92
Smoking started before first birth														
Never smoker	668	204	1 (Reference)					69	1 (Reference)					
No	56	12	0.75	0.36	-	1.57		3	0.43	0.12	-	1.48		
Yes	136	39	0.94	0.59	-	1.50		14	0.93	0.47	-	1.81		
Uncertain	14	4	0.95	0.28	-	3.23		2	1.15	0.23	-	5.63		
Number of cigarettets per day														
Never smoker	668	204	1 (Reference)					69	1 (Reference)					
0 < ≤ 10	139	38	0.93	0.59	-	1.46		11	0.72	0.35	-	1.48		
11 ≤	118	30	0.92	0.56	-	1.50		11	0.87	0.42	-	1.78		
*p for trend*							0.68						0.53	0.75
Duration of smoking (years)														
Never smoker	668	204	1 (Reference)					69	1 (Reference)					
0 < ≤ 21	184	40	0.93	0.60	-	1.46		11	0.71	0.34	-	1.47		
21 <	69	23	0.79	0.45	-	1.39		10	0.94	0.44	-	2.01		
*p for trend*							0.41						0.63	0.93
Pack-years														
Never smoker	668	204	1 (Reference)					69	1 (Reference)					
0 < ≤ 13	165	37	0.82	0.52	-	1.29		9	0.56	0.26	-	1.21		
13 <	86	26	0.98	0.58	-	1.66		11	1.06	0.51	-	2.22		
*p for trend*							0.68						0.69	0.91

All models were adjusted by age (continuous), BMI (< 18.5, 18.5 ≤ < 25.0, 25.0 ≤ < 30.0, 30.0 ≤), occupation (professional or clerical work, industrial work or fishery, agriculture or forestry, other), physical activity (almost no, more than one hour per week), age at menarche (≤ 12, 13, 14, 15 ≤), age at first birth (≤ 24, 25 ≤ ≤ 29, 30 ≤ ≤ 50), family history of breast cancer in mother or sister (yes, no), parity number (0, 1, 2, 3 ≤), use of exogenous female hormone or oral contraceptives (yes, no), referrel status (from screening, other), year of recruitment (continuous), area of residence (Southern Miyagi prefecture, other area) and alcohol drinking (never, current or past).

Table 4 shows the results according to ER+/PgR + and ER-/PgR- status among postmenopausal women. For both subtypes, no association with history of smoking was observed. However, an extremely high risk for ER-/PgR- cancer was found for start of smoking at an early age (OR = 7.01, 95%CI: 2.07-23.73 for ≤19 years), although the confidence interval for this category was wide, and the trend test for age at the start of smoking failed to demonstrate any significance (*P*_trend_ = 0.71). No other exposure variables were associated with breast cancer risk for either of the hormone receptor subtypes.

**Table 4:** **Odds ratio (OR) among postmenopausal women according to smoking status by hormone receptor status**

	Postmenopausal													
	Controls	ER+/PgR+ (n = 366)						ER-/PgR- (n = 195)						***P***_***heterogeneity***_
		Cases	OR	95% CI			p	Cases	OR	95% CI			p	
History of smoking														
Never smoker	1829	327	1 (Reference)					172	1 (Reference)					
Current	161	29	0.96	0.61	-	1.52		14	0.74	0.41	-	1.35		
Past	66	10	0.67	0.33	-	1.38		9	1.25	0.59	-	2.64		
Ever	227	39	0.87	0.58	-	1.29		23	0.88	0.55	-	1.44		0.95
Age at the start of smoking (years)														
Never smoker	1829	327	1 (Reference)					172	1 (Reference)					
26 ≤	113	14	0.69	0.38	-	1.25		10	0.87	0.44	-	1.75		
20 ≤ ≤ 25	72	20	1.30	0.74	-	2.31		5	0.52	0.20	-	1.34		
≤ 19	8	0	-	-	-	-		5	7.01	2.07	-	23.73		
*p for trend*							-						0.71	-
Smoking started before first birth														
Never smoker	1829	327	1 (Reference)					172	1 (Reference)					
No	112	16	0.81	0.46	-	1.44		10	0.83	0.42	-	1.67		
Yes	51	12	1.06	0.53	-	2.10		8	1.17	0.52	-	2.61		
Uncertain	9	2	1.28	0.23	-	7.28		0	-	-	-	-		
Number of cigarettes per day														
Never smoker	1829	327	1 (Reference)					172	1 (Reference)					
0 < ≤ 10	108	16	0.74	0.41	-	1.33		7	0.56	0.25	-	1.26		
11 ≤	88	19	1.14	0.65	-	1.98		13	1.35	0.72	-	2.55		
*p for trend*							0.99						0.79	0.82
Duration of smoking (years)														
Never smoker	1829	327	1 (Reference)					172	1 (Reference)					
0 < ≤ 21	48	7	0.69	0.30	-	1.62		8	1.36	0.61	-	3.03		
21 <	138	25	0.93	0.57	-	1.50		12	0.78	0.41	-	1.49		
*p for trend*							0.62						0.61	0.92
Pack-years														
Never smoker	1829	327	1 (Reference)					172	1 (Reference)					
0 < ≤ 13	65	9	0.67	0.31	-	1.43		6	0.76	0.31	-	1.83		
13 <	118	23	1.01	0.61	-	1.67		14	1.09	0.59	-	2.00		
*p for trend*							0.78						0.94	0.80

All models were adjusted by age (continuous), BMI (< 18.5, 18.5 ≤ < 25.0, 25.0 ≤ < 30.0, 30.0 ≤), occupation (professional or clerical work, industrial work or fishery, agriculture or forestry, other), physical activity (almost no, more than one hour per week), age at menarche (≤ 12, 13, 14, 15 ≤), age at menopause (< 48, 48 ≤ < 51, 51 ≤ < 54, 54 ≤), reson of menopause (natural, others including surgery), age at first birth (≤ 24, 25 ≤ ≤ 29, 30 ≤ ≤ 50), family history of breast cancer in mother or sister (yes, no), parity number (0, 1, 2, 3 ≤), use of exogenous female hormone or oral contraceptives (yes, no), referrel status (from screening, other), year of recruitment (continuous), area of residence (Southern Miyagi prefecture, other area) and alcohol drinking (never, current or past).

Tables 5 and 6 show the association with passive smoking. Overall analysis demonstrated no association between passive smoking and breast cancer risk for any of the four tumor subtypes, although the risk of the ER-/PgR + type may have been uncertain due to the small number of cases (Table 5). On the basis of menopausal status, passive smoking was not associated with either ER+/PgR + or ER-/PgR- tumor type in either pre- or post-menopausal women (Table 6).

**Table 5:** **Odds ratio (OR) according to passive smoking by hormone receptor status**

	Controls	ER+/PgR+ (n = 445)					ER+/PgR- (n = 97)					ER-/PgR+ (n = 16)					ER-/PgR- (n = 215)					***P***_***heterogeneity***_
		Cases	OR	95% CI			Cases	OR	95% CI			Cases	OR	95% CI			Cases	OR	95% CI			ER+/PgR+ vs ER-/PgR-
Passive smoking (husband’s smoking)																						
Never smoker	744	150	1 (Reference)				36	1 (Reference)				7	1 (Reference)				73	1 (Reference)				
Current	826	176	1.13	0.88	-	1.47	36	0.99	0.60	-	1.61	9	1.72	0.56	-	5.27	94	1.17	0.83	-	1.63	
Past	487	119	1.11	0.84	-	1.47	25	0.96	0.56	-	1.65	0	-	-	-	-	48	0.93	0.63	-	1.38	
Ever	1313	295	1.13	0.90	-	1.42	61	0.98	0.63	-	1.51	9	1.01	0.34	-	2.98	142	1.07	0.79	-	1.46	0.78
Analyzed for never-smoking married women.																						

Adjusted by age (continuous), BMI (< 18.5, 18.5 ≤ < 25.0, 25.0 ≤ < 30.0, 30.0 ≤), occupation (professional or clerical work, industrial work or fishery, agriculture or forestry, other), physical activity (almost no, more than one hour per week), menopausal status (premenopausal, postmenopausal), age at menarche (≤ 12, 13, 14, 15 ≤), age at menopause (< 48, 48 ≤ < 51, 51 ≤ < 54, 54 ≤), age at first birth (≤ 24, 25 ≤ ≤ 29, 30 ≤ ≤ 50), family history of breast cancer in mother or sister (yes, no), parity number (0, 1, 2, 3 ≤), use of exogenous female hormone or oral contraceptives (yes, no), referrel status (from screening, other), year of recruitment (continuous), area of residence (Southern Miyagi prefecture, other area) and alcohol drinking (never, current or past).

**Table 6:** **Odds ratio (OR) according to passive smoking by menopausal and hormone receptor status**

	Premenopausal^a^												Postmenopausal^b^											
	Controls	ER+/PgR+ (n = 166)					ER-/PgR- (n = 63)					***P***_heterogeneity_		ER+/PgR+ (n = 262)					ER-/PgR- (n = 137)					***P***_heterogeneity_
		Cases	OR	95% CI			Cases	OR	95% CI				Controls	Cases	OR	95% CI			Cases	OR	95% CI			
Passive smoking (husband’s smoking)																								
Never smoker	158	54	1 (Reference)				21	1 (Reference)					547	90	1 (Reference)				48	1 (Reference)				
Current	288	81	0.96	0.61	-	1.52	35	0.96	0.51	-	1.81		486	85	1.19	0.85	-	1.68	52	1.28	0.83	-	1.97	
Past	105	31	0.76	0.42	-	1.37	7	0.53	0.20	-	1.38		363	87	1.31	0.93	-	1.84	37	1.02	0.64	-	1.63	
Ever	393	112	0.90	0.59	-	1.39	42	0.85	0.46	-	1.56	0.86	849	172	1.25	0.93	-	1.68	89	1.16	0.79	-	1.70	0.74
Analyzed for never-smoking married women.																								

^a^Adjusted by age, BMI, occupation, physical activity, age at menarche, age at first birth, family history of breast cancer in mother or sister, parity number, use of exogenous female hormone or oral contraceptives, referrel status, year of recruitment, area of residence and alcohol drinking.

^b^Adjusted by age, BMI, occupation, physical activity, age at menarche, age at menopause, reson of menopause (natural, others including surgery), age at first birth, family history of breast cancer in mother or sister, parity number, use of exogenous female hormone or oral contraceptives, referrel status, year of recruitment, area of residence and alcohol drinking.

## Discussion

This hospital-based case-control study was designed to investigate the association between smoking and breast cancer risk in relation to joint hormone receptor status. The risks for history of smoking (ever, never) and various smoking-related measures, including age at the start of smoking, whether an individual started smoking before her first birth, the number of cigarettes smoked per day, the duration of smoking, and the number of pack-years, and passive smoking from husbands (ever, never) were evaluated, and analysis based on menopausal status was also performed. Evidence for smoking-related breast cancer risk in relation to hormone receptor subtype has been limited in both Japan and Western countries (Terry and Rohan 2002; Althuis et al. 2004). Therefore, our result is important for helping to clarify the impact of smoking on breast cancer risk.

Although the results from previous studies have been inconsistent with regard to the overall association between smoking and breast cancer risk (Hamajima et al. 2002; Terry and Rohan 2002; Johnson et al. 2011; International Agency for Research on Cancer 2012; Nagata et al. 2006), those related to hormone receptor status have also been inconsistent. Some studies have reported a positive association between ever smoking and the risk of ER + cancer (Yoo et al. 1997; London et al. 1989; Morabia et al. 1998; Gaudet et al. 2013), whereas others have reported a positive association for ER- cancer, or no association (Morabia et al. 1998; Cooper et al. 1989). Although studies focusing on PgR have been few, a cohort study by Manjer et al. found an increased risk of PgR- cancer among ex-smokers and an increased risk of ER-/PgR- cancer among current or ex-smokers (Manjer et al. 2001). In contrast, a case-control study from Japan reported that ever smoking was associated with an increased risk of PgR + cancer (Yoo et al. 1997). A recent cohort study from the US demonstrated a positive association between smoking and the risk of ER+/PgR + cancer among postmenopausal women (Luo et al. 2011).

The present study found that history of smoking (ever, never) had no overall effect on breast cancer risk for any hormone receptor subtype. However, a few smoking-related measures were associated with the risk of ER-/PgR- cancer: the analysis for all subjects showed that having started to smoke at an early age of ≤19 years was associated with an increased risk of ER-/PgR- cancer, although this did not reach statistically significance. According to menopausal status, starting to smoke at an early age of ≤19 years was significantly associated with an increased risk of postmenopausal ER-/PgR- cancer, although there was no linear relationship between age at the start of smoking and the risk of ER-/PgR- cancer. On other hand, no smoking-related measure was found to be associated with the risk of ER+/PgR + cancer in either pre- or postmenopausal women. Although the significant association of an early age at the start of smoking with the risk of postmenopausal ER-/PgR- cancer must be interpreted carefully because of the wide confidence interval, this finding suggests that early exposure to tobacco smoke may stimulate the development of ER-/PgR- cancer. Cell-proliferative activity in the breast is known to be very high during the teenage period (Potten et al. 1988), and therefore during this period breast tissue may be especially susceptible to tobacco smoke (Terry and Rohan 2002; Palmer et al. 1991). Furthermore, detailed analysis of our data found that postmenopausal ER-/PgR- cancer cases and controls who starting smoking at ≤19 years tended to be heavy, long-term smokers (data not shown). Thus, some subjects with particular characteristics who start smoking as teenagers may have a higher risk of developing postmenopausal ER-/PgR- cancer. To our knowledge, previous studies have never evaluated the effect of age at the start of smoking on ER-/PgR cancer risk. To confirm whether age at the start of smoking is indeed related to the risk of ER-/PgR- cancer, further studies including cohort studies are required.

The association of smoking with breast cancer risk observed in previous studies has been explained from the viewpoint of biological mechanisms. First, tobacco smoke contains carcinogenic substances, which may increase the risk of breast cancer regardless of hormone receptor subtype (International Agency for Research on Cancer 2012; Hecht 2002; Luo et al. 2011). Actually, metabolites of tobacco smoke have been detected in the breast fluid or tissues of smokers (Petrakis et al. 1978). However, it has been demonstrated that specific genotypes such as slow N-acetyltransferase 2 genotype affect the enzyme activity for the detoxification of tobacco smoke carcinogens (International Agency for Research on Cancer 2012; Ambrosone et al. 2008; Zhang et al. 2010). The effects of tobacco carcinogens may modified by polymorphism in some genes (International Agency for Research on Cancer 2012; Ambrosone et al. 2008; Zhang et al. 2010; Rabstein et al. 2010; Yang et al. 2007). Second, antiestrogenic effects of smoking may affect the risk of breast cancer (Baron 1984; Tanko and Christiansen 2004; MacMahon et al. 1982). The null association with the risk of receptor-positive breast cancer demonstrated in the present study may have been attributable to such effects. However, the antiestrogenic effects of tobacco smoke on breast cancer risk are not straightforward. A review by Terry et al. indicated that such antiestrogenic effects may be modified by some factors including exogenous female hormone use (Terry and Rohan 2002). The difference in the results between studies conducted in Japan and Western countries may be partly attributable to differences in lifestyles among the study populations. In Japan, far fewer women use exogenous female hormones than in Western countries (IARC Working Group on the Evaluation of Carcinogenic Risks to Humans 2007). Although we adjusted for the use of exogenous female hormones in our present statistical analysis, other unspecified hormone-related factors might have confounded the association between smoking and breast cancer risk.

In relation to the breast cancer risk posed by passive smoking, some previous studies, mainly case-control studies, have indicated that passive smoking is associated with an increased risk of premenopausal breast cancer (Johnson et al. 2011; International Agency for Research on Cancer 2012; Hanaoka et al. 2005). Most cohort studies have found no association for passive smoking (International Agency for Research on Cancer 2012), whereas one cohort study in Japan observed a significant inverse association (Nishino et al. 2001). Although evidence related to hormone receptor subtype have been limited, one case-control study found no association between passive smoking and any of the joint hormone receptor subtypes (Gammon et al. 2004). A recent cohort study from the US indicated that postmenopausal women who had never smoked, but who had been extensively exposed to passive smoking, had a significantly increased risk of breast cancer; however, analysis according to hormone receptor subtype found no significant association with any subtype (Luo et al. 2011). In the present study, in terms of hormone receptor status, no association was observed between passive smoking from husbands and the risk of breast cancer. As there has been some variation in the estimated parameters of passive smoking among previous studies and ours, it may be unreasonable to compare the results directly. However, these studies suggest that the impact of passive smoking on breast cancer risk may not be so large for any tumor subtype.

The present study had both strengths and limitations. In hospital-based case-control studies like ours, some methodological problems are likely to influence the results. First, we considered comparability between the cases and the controls. We selected the controls from among patients admitted to the same hospital as the cases. The participation rates were high for both cases and controls. However, the distribution of risk factors among the control subjects may have differed from that in the general population. To improve comparability between the cases and controls, we excluded patients with disease believed to have been related to smoking from the controls. Consequently, the proportion of ever smokers among the controls (16.6%) was comparable to that in the general population of Miyagi Prefecture (20.5%) (Report on Health Survey in Miyagi. Sendai: Miyagi Prefecture 2005). Additionally, statistical analyses were appropriately controlled for background characteristics such as area of residence and referral status. Although persistent bias might have existed, it is likely that any problems with comparability would have been weakened. Second, it is necessary to consider the possibility of information bias. In particular, some individuals might have changed their smoking habits after contracting diseases, possibly resulting in misclassification of smoking status. However, since the questionnaire was given to each woman on the day of reservation for her first admission to the MCCH before any definite diagnosis or treatment, any recall bias would likely have been minimal. Furthermore, as both cases and controls were selected from among patients admitted to the same hospital, the controls were not healthy subjects. Therefore, even though their smoking habits might have changed, the patterns of change in the cases and controls would have been comparable. Any information bias is unlikely to have distorted our present results. Third, detailed information on passive smoking was not available in the present study. Only information about passive smoking from husbands among married women was included in the analysis. Due to the lack of data on exposure to tobacco smoke during childhood and occupational passive smoking exposure, the role of passive smoking might not have been fully evaluated.

One of the strengths of our study was that breast cancer risk factors such as reproductive factors, BMI and alcohol drinking were controlled for in the analysis. Thus, our study evaluated the independent effects of smoking on breast cancer risk. Some previous studies had not been considered the confounding effects of such risk factors (Palmer and Rosenberg 1993; Nagata et al. 2006; Johnson 2005). Another strength was the low rate of missing data (8.2%) for hormone receptor status. Compared with our present study, the rates of missing data in some previous studies, including cohort studies, were relatively high (Yoo et al. 1997; London et al. 1989).

In conclusion, the present case-control study found no association between history of smoking (ever, never) and breast cancer risk for any hormone receptor subtype. There was also no association between passive smoking (ever, never) from husbands and the risk of breast cancer. However, detailed analysis of smoking-related measures showed that an early age at the start of smoking of ≤19 years was significantly associated with an increased risk of postmenopausal ER-/PgR- cancer, suggesting that early exposure to tobacco smoke may stimulate the development of ER-/PgR- cancer. Other smoking-related measures such as the number of cigarettes smoked per day, the duration of smoking, and the number of pack-years were not associated with breast cancer risk for any hormone receptor subtype. Cigarette smoking, which may be a preventable risk factor, has complicated effects including direct carcinogenic and antiestrogenic effects. Further studies including bigger, pooled cohort studies are required to clarify the association of smoking with breast cancer risk.

## Abbreviations

ER: Estrogen-receptor
PgR: Progesterone-receptor
MCCH: The Miyagi Cancer Center Hospital
OC: Oral contraceptive
EIA: Enzyme immunoassay
IHC: Immunohistochemistry
OR: Odds ratio
CI: Confidence interval
BMI: Body mass index.
